# Supplementary material for: A New Root and Trunk Rot Disease of Grapevine Plantlets Caused by Fusarium in Four Species Complexes
Source: J Fungi (Basel). 2025 Mar 17;11(3):230. doi: 10.3390/jof11030230 (PMC11942937; doi:10.3390/jof11030230)
Supplement: Supplementary file 1 [file jof-11-00230-s001.zip › jof-3497316-supplementary.pdf]

Journal of Fungi

Table S1. Accession numbers of *tef1* from type strains of *Fusarium* species deposited in public databases and used in the phylogenetic analysis.

Species	Type strain	Accession number (*tef1*)
F. fujikuroi	CBS 221.76	MN534010.1
F. annulatum	CBS 258.54	MT010994.1
F. fredkrugeri	CBS 144209	LT996097.1
F. casha	PPRI 21883	MF787261.1
F. chinhoyiense	NRRL 25221	MN534050.1
F. guttiforme	CBS 409.97	MT010999.1
F. vaughaniae	BRIP 76471a	PQ393368.1
F. inflexum	NRRL 20433	AF008479.1
F. landiae	BRIP 76309a	PQ393366.1
F. oxysporum	CBS 144134	MH485044.1
F. triseptatum	CBS 258.50	MH484964.1
F. foetens	CBS 110286	MT011001.1
F. chongqingense	LC 4957	MW620138.1
F. tricinctum	CBS 393.93	AB674263.1
F. brevicaudatum	NRRL 43638	GQ505665.1
F. equiseti	NRRL 26419	GQ505599.1
F. solani	CBS 140079	KT313611.1
F. albidum	CBS 102683	MW834283.1

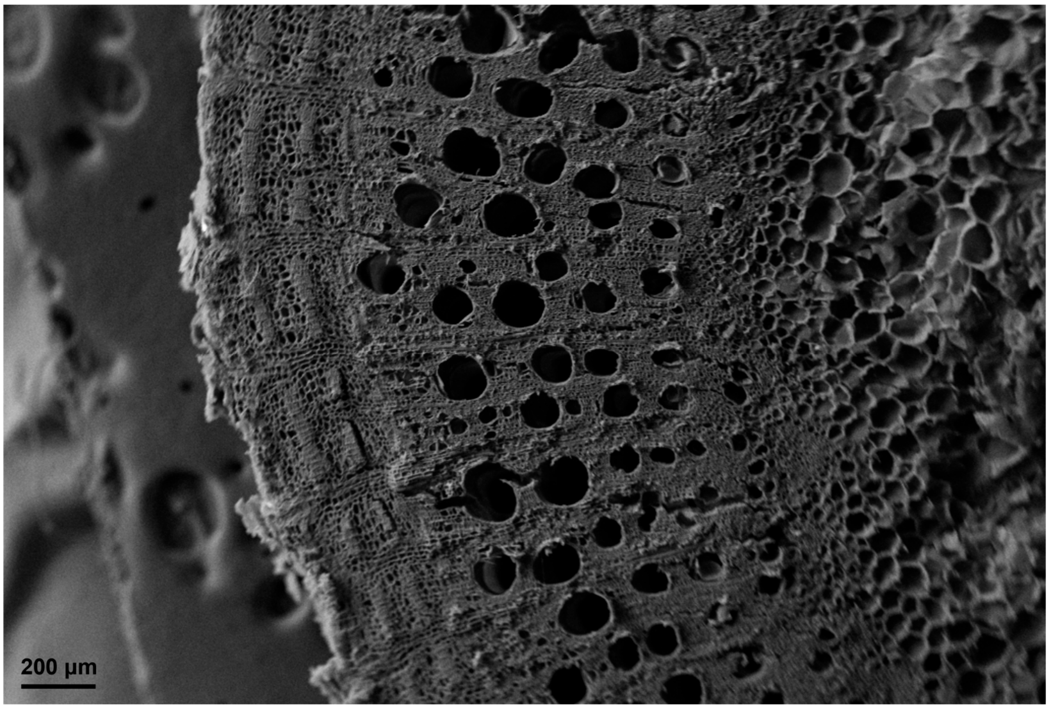

Figure S1. Scanning Electron Microscopy (SEM) image of healthy root vascular tissues of cv. Syrah grafted on IAC766 rootstock. Xylem vessels without obstruction are shown.

 https://doi.org/10.3390/jof11030230
